# Seroprevalence of *Toxoplasma gondii* infection in sheep in Inner Mongolia Province, China

**DOI:** 10.1051/parasite/2020008

**Published:** 2020-02-19

**Authors:** Xinlei Yan, Wenying Han, Yang Wang, Hongbo Zhang, Zhihui Gao

**Affiliations:** 1 Food Science and Engineering College of Inner Mongolia Agricultural University Hohhot 010018 PR China; 2 Inner Mongolia Food Safety and Inspection Testing Center Hohhot 010090 PR China; 3 Inner Mongolia KingGoal Technology Service Co., Ltd. Hohhot 010010 PR China

**Keywords:** *Toxoplasma gondii*, ELISA, Seroprevalence, Sheep, Inner Mongolia, China

## Abstract

*Toxoplasma gondii* is an important zoonotic parasite that can infect almost all warm-blooded animals, including humans, and infection may result in many adverse effects on animal husbandry production. Animal husbandry in Inner Mongolia is well developed, but data on *T. gondii* infection in sheep are lacking. In this study, we determined the seroprevalence and risk factors associated with the seroprevalence of *T. gondii* using an indirect enzyme-linked immunosorbent assay (ELISA) test. A total of 1853 serum samples were collected from 29 counties of Xilin Gol League (*n* = 624), Hohhot City (*n* = 225), Ordos City (*n* = 158), Wulanchabu City (*n* = 144), Bayan Nur City (*n* = 114) and Hulunbeir City (*n* = 588). The overall seroprevalence of *T. gondii* was 15.43%. Risk factor analysis showed that seroprevalence was higher in sheep ≥12 months of age (21.85%) than that in sheep <12 months of age (10.20%) (*p* < 0.01). Seroprevalence was higher in male sheep (18.76%) than females (12.80%) (*p* < 0.01). Barn-feeding sheep (23.13%) had higher prevalence than grazing sheep (10.94%) (*p* < 0.01). The seroprevalence was significantly different in different districts (*p* < 0.01). This study shows that sheep are exposed to *T. gondii* in Inner Mongolia, and provides a data reference for public health and disease control.

## Abstract (fr)

*Toxoplasma gondii* est un parasite zoonotique important qui peut infecter presque tous les animaux à sang chaud, y compris les humains, et son infection peut entraîner de nombreux effets néfastes sur la production animale. L’élevage de Mongolie intérieure est développé, mais les données sur l’infection des moutons par *T. gondii* manquent. Dans cette étude, nous avons déterminé la séroprévalence et les facteurs de risque associés à la séroprévalence de *T. gondii* en utilisant un test immuno-enzymatique indirect (ELISA). Un total de 1853 échantillons de sérum ont été prélevés dans vingt-neuf comtés de la Ligue Xilin Gol (*n* = 624), Hohhot City (*n* = 225), Ordos City (*n* = 158), Wulanchabu City (*n* = 144), Bayan Nur City (*n* = 114) et la ville de Hulunbeir (*n* = 588). La séroprévalence globale de *T. gondii* était de 15,43 %. L’analyse des facteurs de risque a montré que le taux d’infection était plus élevé chez les ovins ≥ 12 mois (21,85 %) que chez les ovins <12 mois (10,20 %) (*p* < 0,01). Les moutons mâles (18,76 %) avaient une séroprévalence plus élevée que les femelles (12,80 %) (*p* < 0,01). Les moutons nourris à l’étable (23,13 %) avaient une séroprévalence plus élevée que ceux au pâturage (10,94 %) (*p* < 0,01). La séroprévalence était significativement différente entre les différents districts (*p* < 0,01). L’étude a montré que les moutons étaient exposés à *T. gondii* en Mongolie intérieure et a fourni une référence de données pour la santé publique et la lutte contre les maladies.

## Introduction

*Toxoplasma gondii* is a food-borne intracellular parasite that can infect nearly all warm-blooded animals worldwide, even humans [9]. Approximately one-third of the human population has been exposed to *T. gondii*. Infection by the parasite may cause cerebral and ocular damage and even death, especially in immunodeficient patients [8, 18]. Humans are mainly infected with *T. gondii* by ingesting uncooked meat and water contaminated by oocysts from the environment, or by vertical transmission [2, 3, 7]. In addition, *T. gondii* can also have a negative influence on animal growth, development and reproduction, and cause great economic loss to livestock husbandry [6]. Livestock become infected mainly by ingesting food and water contaminated with sporulated oocysts [12]. *Toxoplasma gondii* infection in sheep can cause a wide variety of non-specific symptoms (fever and dyspnoea), and specific symptoms (depression, lethargy, vomiting, diarrhea, chorioretinitis, and lymphadenopathy), and can even cause abortions and stillbirths [10]. Recently, increasing consumption of mutton has raised the risk of *T. gondii* infection. Sero-epidemiological surveys have reported a global distribution of *T. gondii* in sheep ranging from less than 4.4% to over 80.0% [5, 14, 19].

As a major livestock husbandry Province in China, the number of sheep stocks in Inner Mongolia has reached over 100 million in recent years, and the animal production economy is one of the most important pillar industries in this district. In the past, many studies have examined the seroprevalence of *T. gondii* infection in livestock, including horses and cattle in Inner Mongolia [16, 21]. However, data on the seroprevalence of *T. gondii* in sheep in Inner Mongolia are not comprehensive nor detailed [4]. The natural grassland of Xilin Gol League and Hulunbeir City is famous throughout the world for its high quality, and sheep production here is prosperous. The population in Hohhot City, Ordos City, Wulanchabu City, and Bayan Nur City accounts for half of the total population in Inner Mongolia. Therefore, samples were collected from these areas, which makes the results more representative. The aim of this study was to determine the current status of the prevalence of *T. gondii* in sheep in Inner Mongolia, and to provide a reference for the prevention and control of *T. gondii*. The results will serve as baseline comparison data for future industrial development and safety assessments, and provide information to public health departments, wildlife managers and researchers.

## Materials and methods

### Serum samples

**Figure 1:**
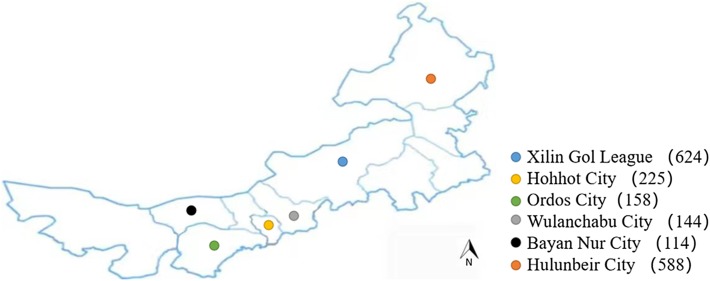
Blood samples were collected from 29 counties of Xilin Gol League, Hohhot City, Ordos City, Wulanchabu City, Bayan Nur City, and Hulunbeir City.

Blood samples were collected from 29 counties of Xilin Gol League, Hohhot City, Ordos City, Wulanchabu City, Bayan Nur City, and Hulunbeir City (Fig. 1) to investigate the presence of serum antibodies against *T. gondii*. A total of 1853 blood samples were selected randomly from September 2018 to November 2019, and the background information of each sample was obtained mainly from the hosts or breeders. No special criteria were applied to different farms. During visits to the localities, blood samples were collected from the jugular vein into a centrifuge tube. These centrifuge tubes filled with blood samples were quickly frozen in a local freezer once collected, and brought back to the laboratory in an incubator. Each of the blood samples was centrifuged at 4000 rpm for 8 min, and serum was separated and stored at −20 °C until further analysis.

### Determination of antibodies against *T. gondii*

Antibodies against *T. gondii* from serum samples were detected by an indirect enzyme-linked immunosorbent assay (ELISA) test, using a commercially available kit (CK-DN74810, 96T), which was obtained from Quanzhou Ruixin Biotechnology Co., Ltd. The detection procedure was carried out in accordance with the protocol described by the manufacturer. When the reaction was complete, the optical density (OD) value was measured at 450 nm using a Microplate Reader within 15 min. Positive and negative controls provided within the kit were included in each test. The serum samples were considered positive if the sample OD value was greater than the cut-off (the cut-off was the sum of the average value of the negative control OD value and 0.15).

### Statistical analysis

Statistical analysis was carried out by chi-square (*χ*^2^) testing with SPSS (Statistical Analysis System, Version 20.0). When *p* < 0.01, the difference was considered extremely significant; when 0.01 < *p* < 0.05, the difference was considered significant; when *p* > 0.05, the difference was not significant. The odds ratio (OR) at the 95% confidence level was used for the determinants influencing the epidemiology of parasites.

## Results

Antibodies against *T. gondii* were found in 286 of the 1853 sheep by the ELISA kit (Table 1); the overall seroprevalence was 15.43%. On the basis of values for *T. gondii* antibody detection, the seroprevalence of four districts was higher than the overall seroprevalence, and Bayan Nur City had the highest seroprevalence (23.68%). Across all districts, Xilin Gol League had the lowest seroprevalence (12.02%). Chi-square test analysis showed that there were significant differences in the prevalence of *T. gondii* infection in different districts (*χ*^2^ = 112.010, *p* value = 0.000) (Table 1).

**Table 1:** Prevalence of *T. gondii* infection in different districts by ELISA.

District	County	No. of examined	No. of positive	Prevalence (%)
Xilin Gol League	East Ujimqin Banner	66	6	9.09
	West Ujimqin Banner	70	8	11.43
	Sonid left Banner	169	34	20.12
	Sonid right Banner	55	7	12.73
	Taibus Banner	70	6	8.57
	Plain and Bordered White Banner	56	5	8.93
	Duolun County	58	5	8.62
	Plain Blue Banner	80	4	5.00
Subtotal		624	75	12.02
Hohhot City	Wuchuan County	32	9	28.13
	Horinger County	27	8	29.63
	Tuoketuo County	29	7	24.13
	Qingshuihe County	70	19	27.14
	Tumd Left Banner	67	10	14.92
Subtotal		225	53	23.56
Ordos City	Dalad Banner	49	12	24.49
	Dongsheng District	46	12	26.09
	Ejin Horo Banner	63	7	11.11
Subtotal		158	31	19.62
Wulanchabu City	Jining District	34	10	29.41
	Chahar Right Back Banner	36	9	25.00
	Siziwang Banner	34	5	14.71
	Chahar Right Middle Banner	40	5	12.50
Subtotal		144	29	20.14
Bayan Nur City	Dengkou County	29	7	24.14
	Hanggin Back Banner	55	12	21.82
	Wuyuan County	30	8	26.67
Subtotal		114	27	23.68
Hulunbeir City	Evenk Autonomous Banner	127	13	10.24
	New Barag Left Banner	113	12	10.62
	Old Barag Banner	92	13	14.13
	Manzhouli City	90	11	12.22
	Yakeshi City	85	10	11.76
	Zalantun City	81	12	14.81
Subtotal		588	71	12.07
Total		1853	286	15.43

In this study, 154 of the 821 male sheep serum samples tested were positive, with a positive rate of 18.76%, and 132 of the 1032 female sheep serum samples tested were positive, with a positive rate of 12.80% (Table 2). There was a significant difference in the seroprevalence of *T. gondii* infection between the sexes (*p <* 0.01). Sheep ≥12 months of age were at higher risk (21.85%, 182/833) than sheep <12 months (10.20%, 104/1020). Barn-feeding sheep were at higher risk (23.13%, 158/683) than grazing sheep (10.94%, 128/1070). There were significant differences in the seroprevalence of *T. gondii* infection in different ages and rearing models (*p <* 0.01). Risk factor analyses showed that sex (OR = 0.682), age (OR = 0.467), and rearing model (OR = 0.473) were risk factors for *T. gondii* infection in sheep (Table 2).

**Table 2:** Prevalence of *T. gondii* infection in different sexes, ages, and rearing models by ELISA.

Factors		No. of tested	No. of positive	Prevalence (%)	*χ*^2^	*p* value	OR (95% CI)
Sex	Male	821	154	18.76	11.500	**0.003**	1
	Female	1032	132	12.80			0.682 (0.531–0.875)
Age	≥12 months	833	182	21.85	34.680	**0.000**	1
	<12 months	1020	104	10.20			0.467 (0.361–0.604)
Rearing model	Barn feeding	683	158	23.13	35.102	**0.000**	1
	Grazing	1170	128	10.94			0.473 (0.368–0.608)

*p* values of statistically significant factors are highlighted in bold.

Over a period of one year, we collected blood samples from different districts every month. Compared with other months, October had the highest prevalence (21.69%, 41/189), and February had the lowest prevalence (10.74%, 13/121). Chi-square test analysis showed that there was no significant difference in the seroprevalence of *T. gondii* infection in different months (*χ*^2^ = 23.157, *p* value = 0.393) (Table 3).

**Table 3:** Seroprevalence of *T. gondii* infection in different months by ELISA.

Month	No. of tested	No. of positive	Prevalence (%)
January	106	12	11.32
February	121	13	10.74
March	130	19	14.62
April	236	31	13.14
May	134	17	12.69
June	104	15	14.42
July	215	39	18.14
August	138	27	19.57
September	223	42	18.83
October	189	41	21.69
November	125	14	11.20
December	132	16	12.12
Total	1853	286	15.43

## Discussion

Antibodies against *T. gondii* were found in 286 out of 1853 sheep (15.43%) in this study, which was higher than that reported in Shandong in 2019 (9.84%) and Yunnan in 2015 (9.70%) [1, 22]. Moreover, Gao et al. reported a prevalence of 17.10% (13/76) for *T. gondii* infection in Chifeng, Inner Mongolia, which was in the same range as the prevalence of infection in this study [4]. In this study, the seroprevalence of *T. gondii* in sheep varied from 5.00% to 29.63% among different counties. There was a great difference in the prevalence of *T. gondii* infection in different districts. We speculated that many factors contributed to this difference, such as climate, elevation, sheep strain, feeding model, and level of disease prevention and control, bearing in mind the vast size of Inner Mongolia Province (1.18 million km^2^). Therefore, in order to make the data more accurate, samples will be collected from more districts in the future.

Moreover, there were significant differences in the seroprevalence of *T. gondii* by sex. Males (18.76%) had a higher risk than females (12.80%) (*p <* 0.01). However, studies in Henan, China, revealed a higher prevalence of *T. gondii* in females than in males [20]. Studies in Yunan, China found no association between sex and the prevalence of *T. gondii* [22]. According to Romanelli et al. the presence of oestrogen in females normally increases immunity, and androgen in males decreases immunity [13]. Therefore, we suspect that sex is likely to work in conjunction with other unknown factors. Moreover, we found that the seroprevalence of *T. gondii* infection had a significant difference concerning age (sheep ≥12 months: 21.85%, and sheep <12 months: 10.20%). A total of 10.20% (104/1020) of sheep <12 months were seropositive. The higher prevalence in sheep ≥12 months was likely due to the prolonged time of exposure and repeated exposure to the oocyst-contaminated environment, resulting in a greater possibility of infection. In this study, there were significant differences in the seroprevalence between barn feeding and grazing rearing systems. Other studies have also shown that barn feeding involves a higher risk than grazing [11]. Since grazing sheep are pastured in comparatively large grazing areas, these sheep are exposed to *T. gondii* oocysts at a low level. However, barn feeding sheep were raised in a concentrated manner, which may increase the chances of *T. gondii* infection among sheep once food, water, or the environment was contaminated by oocysts. This may be the reason why the *T. gondii* infection prevalence in Bayan Nur City was the highest in our study (barn feeding sheep: 114, and no grazing sheep). At the same time, there was no significant difference in the seroprevalence in different months. However, some studies have shown that the seroprevalence is probably related to seasons [17], and the reason for this difference may be due to different environments, temperatures, and various sample qualities. Moreover, studies have shown that high temperatures have little impact on the reduction in viability of *T. gondii* [15]*.*

In this study, seropositive samples were found in 29 counties of all six districts, which suggested that *T. gondii* infection was common in sheep in Inner Mongolia. As an important foodborne zoonotic parasite, *T. gondii* is seriously harmful to people and animals with various routes of infection. Therefore, great attention should be paid to the prevention and control of *T. gondii* in sheep. Certain measures can be taken to reduce the prevalence of *T. gondii* infection in sheep, such as strengthening the management of sheep farms, keeping the barn clean, and preventing feline excreta from polluting sheepfolds, food, or drinking water.

## Conflict of interest

All individual authors declare that they have no conflict of interest (financial, personal, or other).
